# Hydrothermal topotactic epitaxy of SrTiO_3_ on Bi_4_Ti_3_O_12_ nanoplatelets: understanding the interplay of lattice mismatch and supersaturation†

**DOI:** 10.1039/d2na00741j

**Published:** 2023-05-08

**Authors:** Alja Čontala, Nina Daneu, Suraj Gupta, Matjaž Spreitzer, Anton Meden, Marjeta Maček Kržmanc

**Affiliations:** a Advanced Materials Department, Jožef Stefan Institute Jamova Cesta 39 1000 Ljubljana Slovenia marjeta.macek@ijs.si +386 1 477 3292; b Jožef Stefan International Postgraduate School Jamova Cesta 39 1000 Ljubljana Slovenia; c Faculty of Chemistry and Chemical Technology, University of Ljubljana Večna Pot 113 1001 Ljubljana Slovenia

## Abstract

The engineering of epitaxial, two-dimensional (2D) nano-heterostructures has stimulated great interest owing to an expectation of better functional properties (*e.g.*, photocatalytic, piezoelectric). Hydrothermal topotactic epitaxy is one of the promising synthetic approaches for their preparation, particularly the formation of a highly ordered, epitaxial interface and possibilities for the preparation of anisotropic nanostructures of symmetrical materials. The present study highlights the key parameters when steering the alkaline, hydrothermal, topochemical conversion process from Bi_4_Ti_3_O_12_ nanoplatelets to the intermediate, epitaxial, SrTiO_3_/Bi_4_Ti_3_O_12_ nano-heterostructures and the final SrTiO_3_ nanoplatelets by balancing the lattice mismatch and the supersaturation. An atomic-scale examination revealed the formation of an ordered epitaxial SrTiO_3_/Bi_4_Ti_3_O_12_ interface with the presence of dislocations. The SrTiO_3_ grows in islands for a stoichiometric amount of Sr (Sr/Ti = 1) and the growth resembles a layer-by-layer mode for surplus Sr content (Sr/Ti ≥ 12). The latter enables SrTiO_3_ overgrowth of the Bi_4_Ti_3_O_12_ basal surface planes, protecting them against dissolution from the top and consequently ensuring the preservation of the platelet morphology during the entire transformation process, the kinetics of which is controlled by the base concentration. A developed understanding of this particular transformation provides the guiding principles and ideas for designing other defined or complex epitaxial heterostructures and structures under low-temperature hydrothermal conditions.

## Abstract 2

Understanding the interplay between lattice mismatch and supersaturation in the hydrothermal topochemical transformation from Bi_4_Ti_3_O_12_ to SrTiO_3_ nanoplatelets in view of nucleation–crystallization theory and atomic scale interfacial analysis.

## Graphical abstract

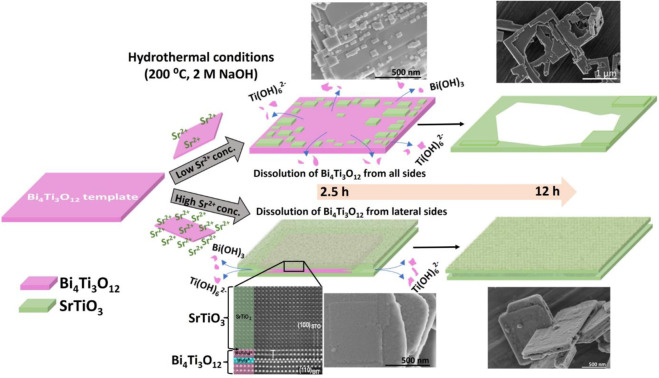

## 1. Introduction

Understanding the reaction mechanisms and nucleation–crystallization phenomena remains one of the most reliable fundamentals for the engineering of nanostructures with enhanced functional characteristics. Thanks to improvements in the resolution of the scanning-transmission electron microscope (STEM), which enables the examination of nanostructures at the atomic level, it is now possible to elucidate the formation mechanisms of even very complex particle architectures. The purpose of the established knowledge is to combine the crystallization principles and exploit them in the design of new, tailored structures or heterostructures. Recently, various heterostructural particles with complex or defined 1-dimensional (1D) or 2-dimensional (2D) morphologies and a tailored spatial composition are attracting increased attention because of their potentially unique or enhanced functional characteristics, which can be implemented in many fields, including photocatalysis and ferroelectric/piezoelectric applications.^1–6^ For example, the construction of effective direct Z-scheme heterostructural photocatalysts by combining two compounds with proper relative band alignments and connected by an epitaxial interface is expected to result in improved photocatalytic performance due to the formation of the interfacial electric field that contributes to the better separation of e^−^–h^+^ pairs and the preservation of a high redox ability.^4^ 2D structures are of particular interest in photocatalysis since their low thickness enables rapid charge transport to the surface and a large 2D area possesses many active sites or provides the maximum face contact when coupling with other photocatalysts in the formation of 2D/2D heterostructures.^1,2^ Considering the enhanced charge mobility at a highly ordered, atomically sharp interface, photocatalysts based on chemically bonded heterostructures with epitaxial contact are expected to show better performance and higher stability compared to physically mixed composite materials.^2,5,6^ These facts were illustrated by the construction of 2D/2D Bi_2_WO_6_/BiOI epitaxial heterostructures by Wang *et al.*,^2^ who demonstrated that this material exhibits improved visible-light photocatalytic activity for the oxidation of NO, compared to that of the individual counterparts. The importance of heterojunction systems is not limited to the application of particulate heterostructures in photocatalysis. Heterostructural films are important constituents of optical devices such as photodetectors and photodiodes.^7–9^ For example, self-powered photodetectors with enhanced performance can be constructed from p–n heterojunctions (p-CuS–ZnS/n-SrTiO_3_), in which photogenerated electron–hole pairs are effectively separated due to a built-in electric field, which results in a stable photocurrent without the need for external power.^7^ Apart from a p–n junction, the built-in ferroelectric field for the improved separation of photo-induced carriers could also be achieved by means of the intrinsic, self-polarization of a ferroelectric film. This was realized in a high-performance UV photodetector based on a mesoporous TiO_2_ layer on ferroelectric BaTiO_3._^8^ A transparent Schottky photodiode constructed from AgNi nanowires (high-work-function electrode), spin-coated on a SrTiO_3_ single crystalline wafer is another example of the advanced architecture of a perovskite-based, multifunctional optical device that enables simultaneous, bias-free, short-wavelength blue-light detection and transparent ultraviolet-light shielding.^9^

When designing heterostructured particles, we must consider that two compounds can form a heteroepitaxial interface under the condition that there is a crystallographic orientation relationship with a sufficiently small lattice mismatch between them.^2^ This natural restriction limits the number of possible material combinations, which are further reduced in the case of the necessary, well-defined 2D or 1D morphologies. Namely, several materials, especially those with a high crystallographic symmetry, do not show a spontaneous tendency for growth in anisotropic 1D or 2D morphologies because these are far from their equilibrium form.^10–12^ One of the strategies to overcome these limitations is offered by the topochemical conversion reactions of 1D or 2D template precursor particles, which in the transformation process convert to new materials with inherited template morphologies. The prerequisite condition for a successful topochemical conversion is that the precursor and objective material are in an orientational relationship or possess similar structural units.^11,12^ Several such kinds of topochemical conversion reactions have been explored in molten-salt media at temperatures between 660 °C and 1100 °C.^11,13–17^ A typical example is the formation of MTiO_3_ (M = Ba, Sr, Pb) perovskite platelets from MBi_4_Ti_4_O_15_ or Bi_4_Ti_3_O_12_ platelets in molten NaCl/KCl in the temperature range 660–1100 °C.^10,13,15,17^

From the energy-consumption standpoint, it would be much more convenient if this type of transformation would be possible in aqueous media at a much lower temperature of hydrothermal synthesis. Different solubilities/stabilities of the precursor/template structure and objective phase in aqueous media, different lattice mismatches with respect to that at high temperature do not guarantee that every confirmed topochemical conversion performed in molten salt could be simply transferred to aqueous media. In the case of good crystallographic matching between the template and the target phase, and their appropriately low solubility in the media, the transformation under hydrothermal conditions is expected to proceed through the epitaxial growth of a new phase on the template. This results in the formation of a clear heteroepitaxial interface between the phases at an intermediate stage of the transformation and the pseudo-morphic replacement of the template by the product after complete conversion.^18–20^ The main advantage of such a hydrothermal topochemical reaction compared to that in molten salt is that negligible diffusion in the solid-state lattice at typical hydrothermal temperatures enables the formation of heterostructures with a well-defined interface. Therefore, by selecting a proper combination of template, its morphology and the target material, we can design versatile, well-defined heterostructures (*e.g.*, 2D/2D) or structures (*e.g.*, 1D, 2D) through a partial or complete hydrothermal topochemical transformation, respectively.

Compared to epitaxial thin-film heterostructures grown by physical vapor deposition, the formation of particulate epitaxial heterostructures from solutions has never been studied in such detail, although the latter requires similar considerations of epitaxy, lattice misfit, defect formation, *etc.* The particle transformation under hydrothermal conditions can be even more complex and sensitive to changes in the conditions, since the template is usually dissolving, while also serving as a substrate for epitaxial growth.^18^ Furthermore, the role and importance of supersaturation is something that must be considered and understood to guide this kind of transformation process.^21^ The current understanding of such a transformation process is limited, and this investigation aims to bridge this knowledge gap through a detailed study of the hydrothermal topochemical transformation of Bi_4_Ti_3_O_12_ platelets to SrTiO_3_ with the shape maintained. Bi_4_Ti_3_O_12_ belongs to the Aurivillius phases, which exhibit a layered structure and are key materials in many fields (piezoelectric, ferroelectric, photocatalysis).^22–25^ SrTiO_3_ with a highly symmetrical crystal structure is an important, multifunctional perovskite material, which depending on doping, oxygen stoichiometry and strain state, can possess versatile characteristics, like microwave dielectric tunability, ferroelectricity, high electronic conductivity, photocatalytic behavior, oxygen conductivity and others.^10^ Recently, SrTiO_3_-based composites have become more significant in photocatalysis, following their use in large-scale hydrogen generation based on solar energy, as demonstrated by the Domen group.^26,27^ Thermodynamically, SrTiO_3_ adopts a cubic perovskite structure and their synthesis with 2D structures is limited. Syntheses of anisometric, 2D SrTiO_3_ crystallites were mainly initiated by the use of SrTiO_3_ platelets for the fabrication of preferentially oriented, textured ceramics. In addition to 2D structures of Sr_3_Ti_2_O_7_ and SrBi_4_Ti_4_O_15_, Bi_4_Ti_3_O_12_ platelets were also shown to be an appropriate template precursor for the preparation of SrTiO_3_ platelets through a topochemical conversion reaction, which until recently was solely studied in molten-salt (NaCl/KCl) media at 1050–1200 °C.^10,16^ In analogy with the already-reported topochemical conversion of Bi_4_Ti_3_O_12_ platelets into SrTiO_3_ platelets in molten salt at 1100 °C,^16^ our group was the first to design the hydrothermal conditions for the Bi_4_Ti_3_O_12_ nanoplatelets to transfer to SrTiO_3_ nanostructures with a preserved 2D-platelet morphology at a much lower temperature of 200 °C.^28^ We also showed that the SrTiO_3_/Bi_4_Ti_3_O_12_ heterostructural platelets that formed at an intermediate stage of transformation have a promising photocatalytic H_2_ evolution, 15-times more than commercial SrTiO_3_ nanopowders.^29^ We disclosed that the transformation from Bi_4_Ti_3_O_12_ to SrTiO_3_ platelets proceeds through the dissolution of Bi_4_Ti_3_O_12_ and the epitaxial growth of SrTiO_3_ over the basal surface planes of the template with the formation of the epitaxial SrTiO_3_/Bi_4_Ti_3_O_12_ heterostructures and SrTiO_3_ platelets at the intermediate and completed transformation stages, respectively. In the present study we gain further unprecedented insights into this transformation process through an atomic-scale examination of the SrTiO_3_/Bi_4_Ti_3_O_12_ interface, an assessment of the relevant lattice misfits and an evaluation of the role and importance of supersaturation in controlling the nucleation and crystallization processes and consequently the transformation pathway. This investigation is one of the few fundamental studies on understanding the formation mechanism of epitaxial, heterostructural SrTiO_3_/Bi_4_Ti_3_O_12_ and SrTiO_3_ platelets that will pave the way for the design of other epitaxial nano-heterostructures and anisotropic nanostructures under hydrothermal conditions.

## 2. Experimental

### 2.1. Chemicals

All chemicals were of analytical grade and were used as received without further purification. KCl (Sigma-Aldrich, ≥99.0%), NaCl (Merck, ≥99.7%), Bi_2_O_3_ nanopowder (Alfa Aesar, 99.8%), TiO_2_ (P25, Degussa), HNO_3_ (VWR, 68%), SrCl_2_·6H_2_O (Sigma-Aldrich, ≥99.0%), NaOH (Fisher Chemicals, ≥98.7%). The water used for the study was purified with a system to produce 18.2 MΩ cm ultra-pure water (Purelab Option-Q7, ELGA).

### 2.2. Synthesis of Bi_4_Ti_3_O_12_ template platelets

Bi_4_Ti_3_O_12_ template nanoplates were synthesized by a molten-salt method from KCl/NaCl salt, Bi_2_O_3_ nanopowder, and TiO_2_ nanopowder (0.500 g), so that the molar ratio of NaCl : KCl : Bi_2_O_3_ : TiO_2_ was 50 : 50 : 2 : 3.^25,28,29^ Heating and cooling rates were 10 °C min^−1^ and the reaction temperature was 800 °C with a holding time of 2 h. Afterwards, Bi_4_Ti_3_O_12_ particles were washed with deionized water to remove the salt, 2 mol L^−1^ HNO_3_ to remove the secondary phases, and finally, once again with deionized water to ensure a neutral pH.^13^ The product particles were freeze-dried under vacuum conditions.

### 2.3. Transformation of Bi_4_Ti_3_O_12_ into SrTiO_3_/Bi_4_Ti_3_O_12_ and SrTiO_3_

The transformation reactions were performed under stirring hydrothermal conditions in a Berghof high-pressure reactor with a Teflon (PTFE) insert. In a typical procedure, Bi_4_Ti_3_O_12_ platelets were admixed (0.057 mol L^−1^) into a water solution containing dissolved SrCl_2_·6H_2_O. The suspension was sonicated for 10 minutes. Afterwards, the suspension was quantitatively transferred to a PTFE-lined insert and subsequently a concentrated NaOH solution was added. Both the NaOH solution and the dispersion of Bi_4_Ti_3_O_12_ platelets were cooled down to room temperature (25 °C) before mixing. Finally, deionized water was added to the line mark in the PTFE insert to achieve a 70% filling. The concentrations of the reagents in the precursor suspension before the hydrothermal reactions are presented in Table S1.† The concentration of Bi_4_Ti_3_O_12_ was the same (*c*_Bi_4_Ti_3_O_12__ = 0.00102 mol L^−1^) in all experiments, while the concentration of the NaOH (*c*_NaOH_) solution was 2 mol L^−1^ or 6 mol L^−1^ and the concentration of SrCl_2_·6H_2_O varied from 0.00306 mol L^−1^ to 0.07344 mol L^−1^, corresponding to a variation of the initial Sr/Ti molar ratios from 1 : 1 to 24 : 1. The initial Sr/Ti ratio refers to the molar ratio of the added strontium precursor to the overall Ti in Bi_4_Ti_3_O_12_. No other titanium compound was added. The reactions were performed under stirring conditions at 200 °C with a duration from 1 hour to 12 hours. After the reaction, the system was cooled naturally. The product was washed with water by centrifugation (until pH = 7), soaked in 30 mL of 1 mol L^−1^ HNO_3_ for 5 min, and washed again with water (again until pH = 7). In the end, the washed product was freeze-dried to obtain a fine powder product.

### 2.4. Characterization

X-ray powder diffraction and X-ray diffraction (XRD) for the platelets cast on a single-crystalline silicon substrate were collected with an X-ray diffractometer (Empyrean, Malvern PANalytical) with CuKα_1_ radiation (*λ* = 1.5406 Å). The percentages of SrTiO_3_ in the SrTiO_3_/Bi_4_Ti_3_O_12_ heterostructures were estimated based on the intensity ratios of the (200) diffraction of SrTiO_3_ to (008) and (0014) diffractions of Bi_4_Ti_3_O_12_ to evaluate the progress of the transformation. These calculations were performed from the XRD patterns of the platelets cast on single-crystalline silicon substrates. The average weight percentages of SrTiO_3_ in the synthesized samples were inferred from the calibration curve, which was made based on XRD patterns of the mixtures of SrTiO_3_ and Bi_4_Ti_3_O_12_ platelets with known weight ratios of both components. The XRD measurements for the calibration curve were also performed for the platelets cast on the single-crystalline silicon substrate. Using this method, the majority of the platelets were preferentially oriented parallel to the sample holder (single-crystalline silicon), thus giving reproducible intensities of the (008) and (0014) diffractions of Bi_4_Ti_3_O_12_ and (200) diffractions of SrTiO_3_, that are parallel to the substrate in this platelet orientation.

The morphologies of the platelets were examined with a scanning electron microscope (SEM) (Schottky FEG, Verios HP 4G, Termo Fischer, USA) operated at 2 kV and 13 pA with a beam deceleration (2 kV) as well as a probe Cs-corrected scanning-transmission electron microscope (STEM Jeol ARM 200 CF, JEOL, Japan) operated at 200 kV.

STEM investigations were performed on the platelets from the top-down and the cross-sectional (edge on) views. For the top-down examinations, powder samples were dispersed in absolute ethanol, sonicated for 15 minutes and a droplet of dispersion was applied to the lacey carbon-coated copper grid. As-prepared platelets spontaneously align with their largest surface parallel to the carbon grid. Any further thinning was not necessary in this case. For edge-on observations of the platelets in cross-section, a small amount of sample powder was mixed together with epoxy resin for the preparation of lamellae. The electron transparency of the lamellae was achieved by mechanical polishing and ion milling in an automatic tripod polisher (Gatan PIPS Model 691, USA). The samples were coated with 2 nm of carbon before STEM observation (PECS, Model 682, USA).

## 3. Results and discussion

### 3.1. Theoretical and experimental background for controlling Bi_4_Ti_3_O_12_-to-SrTiO_3_ transformation under hydrothermal conditions

The prerequisite condition for hydrothermal topochemical conversion is good crystallographic matching between the template and the precipitating new phase, which enables a high nucleation rate and epitaxial growth of the latter on the template, which dissolves through the advancement of the process and as a result, the precursor phase is progressively replaced by the new phase.^18,19^ Therefore, to control the coupling of template dissolution and epitaxial growth, it is important to know the characteristics of the template and its dissolution in the media, as well as to understand all the solution and interfacial processes. In the presented Bi_4_Ti_3_O_12_-to-SrTiO_3_ transformation, detailed considerations of these parameters are given below.

**Fig. 1:**
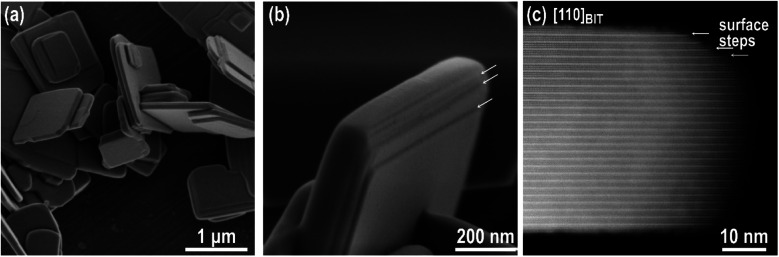
(a) SEM micrograph of Bi_4_Ti_3_O_12_ platelets. (b) Magnified SEM image of Bi_4_Ti_3_O_12_ platelet with growth steps (arrows) parallel to the basal planes. (c) Atomic-scale STEM image of a Bi_4_Ti_3_O_12_ platelet oriented along the [110]_BIT_ zone axis with lateral surface steps.

The Bi_4_Ti_3_O_12_ template platelets that were subject to the Bi_4_Ti_3_O_12_-to-SrTiO_3_ transformation studies exhibited an average side length of 1–2 μm and thickness of 50–100 nm. These were typical dimensions of the platelets grown in molten NaCl/KCl salt at 800 °C for 2 hours (Fig. 1).^28,29^ The STEM examination of the Bi_4_Ti_3_O_12_ platelets confirmed their layered structure, with alternation of the pseudo-perovskite ([Bi_2_Ti_3_O_10_]^2−^) blocks and the bismuth oxide ([Bi_2_O_2_]^2+^) layers, whereby the latter exclusively terminated the basal surface planes of the as-prepared platelets, while both types of structural units were exposed at the lateral surface (Fig. 1c). According to the STEM analysis, typical platelets contain few or no planar defects and have atomically flat, basal surface planes. Steps are more frequently present close to the platelet's edge and at the lateral surfaces (Fig. 1b and c). Such a morphological development stems from the anisotropic layer-by-layer growth of the Bi_4_Ti_3_O_12_ platelets during their synthesis in molten salt.

The mechanism of the hydrothermal conversion from Bi_4_Ti_3_O_12_ platelets to SrTiO_3_ platelets in alkaline media under high supersaturation conditions is described in detail in our previous study^29^ and briefly summarized below. The transformation process proceeded through two main chemical reactions: the dissolution of Bi_4_Ti_3_O_12_ according to eqn (1) and the crystallization of SrTiO_3_, expressed by eqn (2).^18,29^

Dissolution of Bi_4_Ti_3_O_12_:1
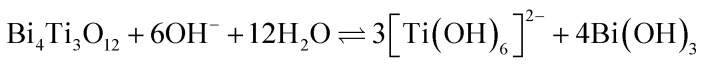


Crystallization of SrTiO_3_:

2 [Ti(OH)_6_]^2−^(aq) + Sr^2+^(aq) → SrTiO_3_(s) + 3H_2_O

Bi_4_Ti_3_O_12_ platelets are the source of [Ti(OH)_6_]^2−^(aq) and the substrate for the epitaxial growth of SrTiO_3_. There is no other source of [Ti(OH)_6_]^2−^(aq) since no other titanium compound was present in the system. Matching of the phases in [100]_STO_ (010)_STO_ II [110]_BIT_ (11̄0)_BIT_ and [11̄0]_STO_ (110)_STO_ II [100]_BIT_ (200)_BIT_ orientational relationships dictates the (100) SrTiO_3_ growth on the basal surface planes of (001)-oriented Bi_4_Ti_3_O_12_ platelets. Control over the process can be exerted based on a consideration of classical nucleation theory and eqn (3), which defines that the nucleation barrier (Δ*g*_n_) is proportional to the third power of the interfacial free energy (*α*) and inversely proportional to the square of the natural logarithm of supersaturation (*S*) ((ln *S*)^2^).^21,30,31^ In the case of highly crystalline Bi_4_Ti_3_O_12_ platelets, the major contribution to the interfacial free energy comes from the lattice misfits of the Bi_4_Ti_3_O_12_ and SrTiO_3_ pairs of lattice planes from above-mentioned orientational relationships. In the studied system, supersaturation (eqn (4) is defined as the ratio between the product of activities of the dissolved species ([Ti(OH)_6_)]^2−^(aq) and Sr^2+^(aq)) immediately before the SrTiO_3_ formation and the thermodynamic solubility product (*K*_s_) of SrTiO_3_.^18,29^3
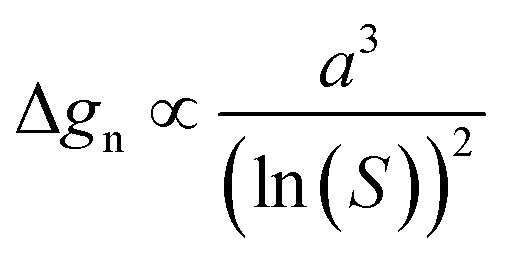
4
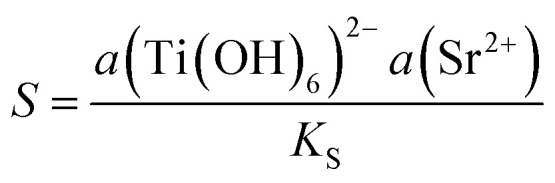


In accordance with eqn (3) and (4), higher concentrations of [Ti(OH)_6_]^2−^(aq) and Sr^2+^(aq) (*i.e.* higher supersaturation) promote the nucleation of SrTiO_3_ on Bi_4_Ti_3_O_12_. In our previous study, a high supersaturation condition was achieved by using 12-times more Sr than needed for the stoichiometric transformation and a high concentration of NaOH (6 mol L^−1^), which provides a sufficiently high concentration of [Ti(OH)_6_]^2−^(aq) (eqn (1)) through the dissolution of pseudo-perovskite layers of the Bi_4_Ti_3_O_12_ platelet (Fig. S1, ESI†). These empirically determined, high supersaturation conditions, enable a low nucleation-energy barrier (eqn (3)), resulting in a high nucleation rate of SrTiO_3_ on the basal surface planes of the Bi_4_Ti_3_O_12_ platelets, with the formation of the SrTiO_3_ protective layer, which restricts Bi_4_Ti_3_O_12_ dissolution mainly from the lateral sides. This results in the formation of a groove, which deepens with the progress of the reaction, while the epitaxial SrTiO_3_ layers on both basal surfaces thicken (Fig. S1, ESI†).^29^ When the Bi_4_Ti_3_O_12_ inside the groove dissolves to the last perovskite layer, SrTiO_3_ also starts to grow epitaxially from the inner side. This is inferred based on the captured monoatomic bismuth layer that extends along the completely converted platelet part.^29^ So, the reaction proceeds from Bi_4_Ti_3_O_12_*via* the SrTiO_3_/Bi_4_Ti_3_O_12_ epitaxial heterostructure until a complete pseudo-morphic transformation to (100)-oriented SrTiO_3_ with the preservation of the overall platelet morphology through the entire conversion process. The final SrTiO_3_ platelets, which consist of two parallel, partially intergrown platelets, reflect the transformation process (dissolution of Bi_4_Ti_3_O_12_ from the lateral side and epitaxial growth of SrTiO_3_ on both basal surface planes (Fig. S1, ESI†)). With these initial ideas, we gain new mechanistic insights into the transformation process, by empirically probing the role of different factors in eqn (3), such as the interfacial free energy (*α*) and supersaturation (*S*).

### 3.2. Role of the interfacial free energy

The interfacial free energy (*α*) in the numerator of eqn (3), is a combination of the energy due to the difference in chemical bonding at the interface and the strain energy due to incoherent ordering at the interface. These characteristics comprise lattice mismatch. In general, interfacial energy (*α*) is lower when the two phases (*i.e.*, epitaxial layer and substrate) are crystallographically well aligned and coherent.^21,32^ The misfits in our system were evaluated for the [100]_STO_ (010)_STO_ II [110]_BIT_ (11̄0)_BIT_ and [11̄0]_STO_ (110)_STO_ II [100]_BIT_ (200)_BIT_ orientational relationships, while the unit-cell parameters and relevant lattice spacings were obtained from the Rietveld refinement of the XRD patterns of SrTiO_3_ and Bi_4_Ti_3_O_12_ at room temperature (RT) and 200 °C (Tables 1 and 2). The observed increase in the unit-cell parameters of Bi_4_Ti_3_O_12_ (∼0.12% to 0.23%) and SrTiO_3_ (∼0.17%) at 200 °C, compared to RT, is in good agreement with the calculated temperature coefficient of expansion (TCE) reported in the literature (Table 1).^33,34^ Based on the comparison of the TCE of both materials we can assume that the thermal misfit is negligible. From the unit-cell parameters, the inferred distance of the (110) plane in Bi_4_Ti_3_O_12_ is 3.8417 Å and 3.8475 Å, while that of the (100) plane in SrTiO_3_ is 3.910 Å and 3.9171 Å at RT and 200 °C, respectively (Table 2). The lattice mismatch between the (110) plane in Bi_4_Ti_3_O_12_ and (100) in SrTiO_3_ is 1.79% and does not change significantly in the studied temperature range. The (020) plane in Bi_4_Ti_3_O_12_ and (110) in SrTiO_3_ exhibit the largest misfit (2.1%) among the relevant pairs of lattice planes. The lattice misfits, which vary from 1.4% to 2.1%, are shown in Table 2, represent unavoidable contributions to the interfacial free energy (eqn (3)) for the growth of the SrTiO_3_ on the basal surface plane of the Bi_4_Ti_3_O_12_ platelet. In general, in the process of epitaxial growth, the misfit can be eliminated/reduced by elastic strain or accommodated by misfit dislocations. The latter are formed when the misfit is too large to be completely eliminated by elastic strain.^35^

**Table 1:** Unit-cell parameters of Bi_4_Ti_3_O_12_ and SrTiO_3_ obtained from the Rietveld structural refinement. Experimentally determined expansion of the unit-cell parameters between room-temperature (RT) and 200 °C, presented in % and as thermal expansion coefficients (TCEs) and their comparison with the reported TCEs

Material		25 °C	200 °C	Expansion (%)	TCE (RT–200 °C) (K^−1^)		
					This study	Average	Literature^33,34^
Bi_4_Ti_3_O_12_	*a*	5.4517(5) Å	5.4584(5) Å	0.12	7.0 × 10^−6^	1.0 × 10^−5^	(1.3 ± 0.2) × 10^−5^
	*b*	5.4143(5) Å	5.4239(5) Å	0.18	1.0 × 10^−5^		
	*c*	32.796(2) Å	32.872(2) Å	0.23	1.3 × 10^−5^		
SrTiO_3_	*a*	3.9105(1) Å	3.9171(2) Å	0.17	2.9 × 10^−5^	—	3.2 × 10^−5^

**Table 2:** Misfits between Bi_4_Ti_3_O_12_ (BIT) and SrTiO_3_ (STO) for different pairs of lattice planes, as observed in Fig. 2 and calculated from the experimentally determined unit-cell parameters at room-temperature (RT) and at 200 °C. The difference between [100] and [010] in Bi_4_Ti_3_O_12_ is too small to be resolved by STEM

	(110)_BIT_–(010)_STO_		(200)_BIT_–(110)_STO_		(020)_BIT_–(110)_STO_	
	RT	200 °C	RT	200 °C	RT	200 °C
*d*_BIT_, Å	3.8417	3.8475	2.72585	2.729	2.7072	2.712
*d*_STO_, Å	3.9105	3.9171	2.7651	2.7698	2.7651	2.7698
Misfit, %	1.77	1.79	1.43	1.48	2.12	2.11
Period of misfit, Å	222	220	195	189	144	133
Period of misfit, lattice planes	57	56	70	68	52	48

**Fig. 2:**
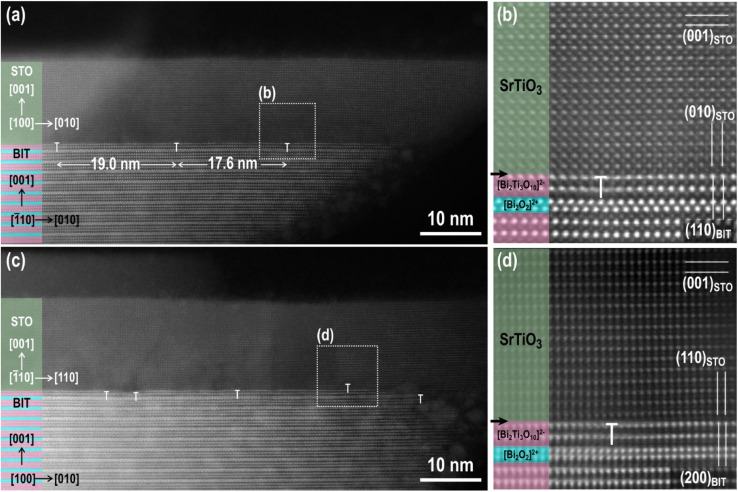
(a–d) Cross-sectional HAADF-HR-STEM image of the SrTiO_3_/Bi_4_Ti_3_O_12_ interface: (a and b) [100]_STO_ (010)_STO_ II [110]_BIT_ (11̄0)_BIT_ and (c and d) [11̄0]_STO_ (110)_STO_ II [100]_BIT_ (200)_BIT_, HR-STEM micrographs in (b) and (d) showing the magnified areas denoted in (a) and (c), respectively. SrTiO_3_/Bi_4_Ti_3_O_12_ platelets, presented in micrographs (a)–(d), were synthesized at 200 °C for 2.5 hours in 6 mol L^−1^ NaOH and at Sr/Ti = 12. In the (a)–(d) the [Bi_2_O_2_]^2+^ layers and pseudo-perovskite [Bi_2_Ti_3_O_10_]^2−^ blocks are marked with blue and pink, respectively.

To evaluate how the calculated misfits (Table 2) are manifested across the SrTiO_3_/Bi_4_Ti_3_O_12_ interface, we performed a high-angle annular darkfield (HAADF) STEM analysis of the interface in partially transformed platelets (after 2.5 h at 200 °C in 6 mol L^−1^ NaOH with Sr/Ti = 12). The results are shown in Fig. 2. The images reveal that SrTiO_3_ grows epitaxially on the pseudo-perovskite layer of the Bi_4_Ti_3_O_12_ substrate and not on the [Bi_2_O_2_]^2−^ layer, which is the termination layer of the as-prepared Bi_4_Ti_3_O_12_ platelets.^29^ We analyzed the Bi_4_Ti_3_O_12_ platelets before and after exposure to NaOH at 200 °C for 1 hour and confirmed that the latter treatment removes the surface [Bi_2_O_2_]^2−^ layer (Fig. S2, ESI†). We believe that the removal of the bismuth oxide termination layer facilitates the nucleation of SrTiO_3_ on the pseudo-perovskite-terminated surface of the Bi_4_Ti_3_O_12_ platelets and enhances the development of a coherent hetero-interface, as observed in high-resolution (HR) HAADF-STEM images in Fig. 2a–d. The images reveal that the misfit between the underlying Bi_4_Ti_3_O_12_ substrate and the larger-unit-cell-SrTiO_3_ layer is compensated by missing lattice planes in the SrTiO_3_ film. In an unstrained film, the average spacing between these misfit dislocations is calculated from the lattice spacings between the substrate (s) and the film (f): *x*_0_ = *a*_f_/(*a*_f_ − *a*_s_), where *x*_0_ represents the period of the misfit (number of lattice planes between two adjacent dislocations).^36,37^ In our calculations *a*_f_ represents the relevant lattice spacings of SrTiO_3_ (*i.e.* (010)_STO_ and (110)_STO_), while *a*_s_ are the corresponding lattice spacings of Bi_4_Ti_3_O_12_ (*i.e.*, (110)_BIT_, (200)_BIT_ and (020)_BIT_). The calculated periods of misfit-dislocations (in Å and in the number of lattice planes) for various pairs of lattice planes are shown in Table 2. The expected number of planes separating two dislocations in the SrTiO_3_ film on the Bi_4_Ti_3_O_12_ substrate with the [100]_STO_ (010)_STO_ II [11̄0]_BIT_ (110)_BIT_ orientational relationship is between 56 and 57 (010)_STO_ lattice planes, corresponding to approximately 22 nm. The calculated distances are slightly larger than the spacings between the dislocations observed in the experimental image (Fig. 2a). The spacings between the misfit dislocations in the other investigated low-index zone axis with [11̄0]_STO_ (110)_STO_ II [010]_BIT_ (200)_BIT_ (Fig. 2c) are irregular and depend on the sample cross-section.

Magnified images of the areas around the misfit dislocations (Fig. 2b and d) show a locally, slightly fuzzy contrast, which indicates that the crystal structure of the last pseudo-perovskite layer of the Bi_4_Ti_3_O_12_ is slightly disturbed around the dislocation cores due to the strain imposed on the substrate by the SrTiO_3_ layer with the larger unit cell. On the other hand, the SrTiO_3_ layer seems to be relaxed after a few atomic layers.

Based on the HAADF HR STEM micrographs (Fig. 2b and d) we believe that the SrTiO_3_ growth starts with the deposition of Sr^2+^ ions, followed by the deposition of the perovskite TiO_6_^2−^ octahedra. The growth continues with the next layer of Sr^2+^ and so on. This atomic-scale insight at the SrTiO_3_/Bi_4_Ti_3_O_12_ interface could also be one of the reasons why higher Sr/Ti ratios also play such a beneficial role in the preservation of the platelet morphology during the transformation process. Due to the competition between the template dissolution and the epitaxial growth process, the rapid deposition of the first layer of Sr^2+^ ions over the naked pseudo-perovskite-terminated basal surface plane is important for the protection of the platelet against dissolution from the top and also for the continuation of the epitaxial growth. Higher Sr^2+^(aq) concentrations (*c*_Sr^2+^_) accelerate the diffusion of the Sr^2+^ ions to the surface, facilitating the formation of the first and subsequent Sr^2+^ layers.

**Fig. 3:**
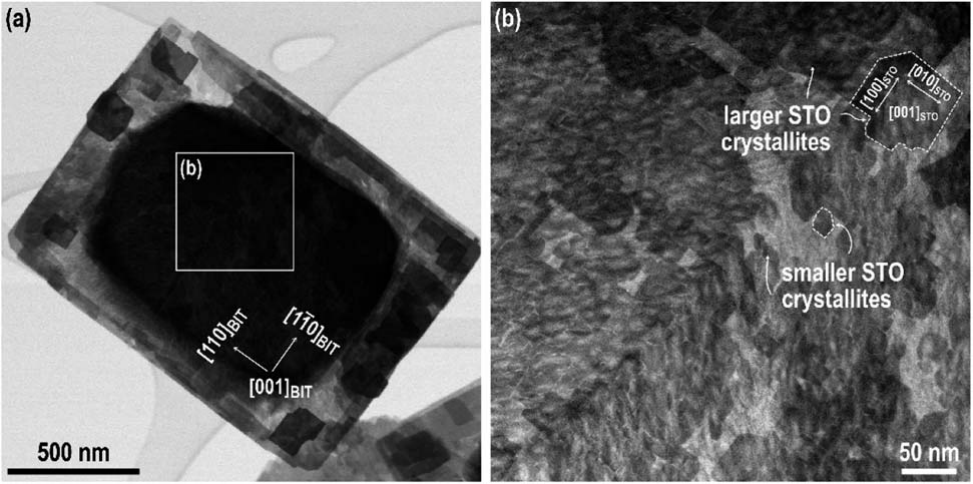
(a) Bright-field (BF) STEM image of the SrTiO_3_/Bi_4_Ti_3_O_12_ platelet, prepared at 200 °C for 1 hour at Sr/Ti = 12 in 6 mol L^−1^ NaOH. (b) Magnified top-down dark field (DF) STEM image of the marked area, shown in (a) reveals that the top SrTiO_3_ (STO) layer is composed of domains.

Additional information about the growth mechanism was obtained from the top-down STEM examination of the SrTiO_3_/Bi_4_Ti_3_O_12_ platelets after a short conversion time (200 °C/1 hour, Sr/Ti = 12, 6 mol L^−1^ NaOH) (Fig. 3). It can be seen from Fig. 3b that in the first layers (thickness of 1–2 structural units) SrTiO_3_ epitaxial growth occurs with the formation of crystallite islands. The size of the crystallites is limited by the formation of misfit dislocations along the two perpendicular directions [1̄10]_BIT_ II [010]_STO_ and [110]_BIT_ II [100]_STO_ (Fig. 3). After 1–2 unit cells the crystallites appear to be larger (Fig. 3b). This is in accordance with the cross-sectional STEM observation (Fig. 2b and d), which reveals that the influence of dislocations vanishes after two structural units.

### 3.3. Role of supersaturation

In general, the degree of supersaturation determines the nucleation-and-growth mechanism, and therefore its control is extremely important in the tailoring of the crystallization particle morphologies^31^ and thin-film growth of functional materials from solutions.^38,39^ Similarly, in the transformation from Bi_4_Ti_3_O_12_ to SrTiO_3,_ according to eqn (3), the nucleation-and-crystallization landscape can be governed by supersaturation. For this reason, the hydrothermal reactions were performed under conditions with different amounts of strontium (1 ≤ Sr/Ti ≤ 24) and NaOH concentrations. Such an experimental plan was made with the assumption that NaOH controls the rate of Bi_4_Ti_3_O_12_ dissolution and consequently determines the concentration of Ti(OH)_6_^2−^, while for the concentration of Sr^2+^, we speculate that larger amounts of strontium render more dissolved Sr^2+^, which can participate in the formation of SrTiO_3_. Theoretically, it is possible to predict the equilibrium concentrations of aqueous species (supersaturation) with thermodynamic modelling. In the literature, this was already performed for the formation of SrTiO_3_ from simple TiO_2_-based precursors (anatase, rutile, hydrous TiO_2_·*x*H_2_O gel) and strontium precursors (Sr(NO_3_)_2_, Sr(OH)_2_·8H_2_O).^18,40,41^ However, for the present system, dealing with the transformation from Bi_4_Ti_3_O_12_ to SrTiO_3,_ any accurate thermodynamic modelling is hampered by the complexity of the system arising from several possible side reactions (formation of other bismuth titanium compounds (*e.g.* Bi_12_TiO_20_)), the ill-understood chemistry of Bi(OH)_3_ and accordingly the lack of reliable thermodynamic data. For this reason, the supersaturation in this study was considered at the empirical level. Based on the observed morphological development and our understanding of the general effects of supersaturation on nucleation and growth, it is possible to make a rough comparison of the supersaturations under different conditions. The smallest selected Sr/Ti ratio was 1, because at least a stoichiometric Sr/Ti ratio is needed for a complete transformation from Bi_4_Ti_3_O_12_ to SrTiO_3_. Systematic studies were also performed for Sr^2+^(aq) concentrations that significantly exceed the stoichiometric ratio (*i.e.*, Sr/Ti = 3, 12, 24), while NaOH concentrations were varied from 2 mol L^−1^ to 6 mol L^−1^. The growth and phase compositions were examined after 2.5 and 12 h.

#### 3.3.1. Transformation control at a lower base concentration (2 mol L^−1^ NaOH)

**Fig. 4:**
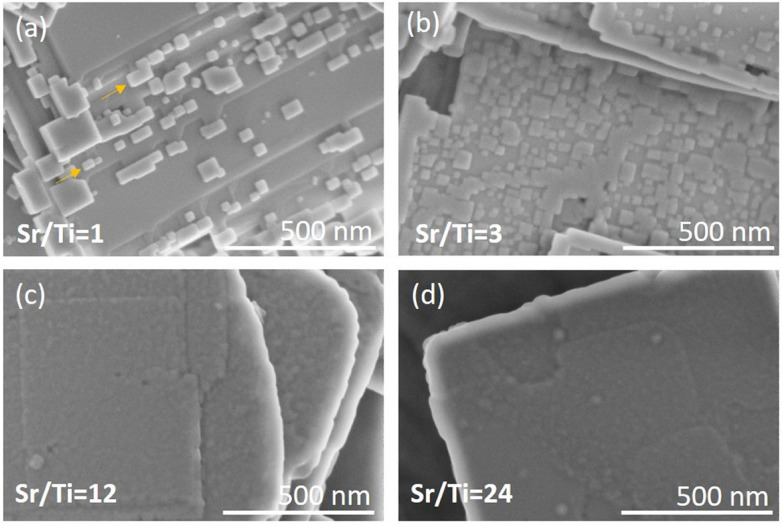
SEM micrographs of the part of the SrTiO_3_/Bi_4_Ti_3_O_12_ platelet after 2.5 h at 200 °C at different Sr/Ti ratios in 2 mol L^−1^ NaOH: (a) Sr/Ti = 1, (b) Sr/Ti = 3, (c) Sr/Ti = 12, (d) Sr/Ti = 24. The arrows mark some of the SrTiO_3_ islands, growing in line.

Fig. 4a and S3, ESI,† show the typical growth mode of SrTiO_3_ on Bi_4_Ti_3_O_12_ after 2.5 h of hydrothermal reaction at Sr/Ti = 1 and in 2 mol L^−1^ NaOH. Based on this lower NaOH concentration, compared to the 6 mol L^−1^ NaOH in our previous study,^29^ a slower dissolution rate of Bi_4_Ti_3_O_12_ and consequently a lower concentration of Ti(OH)_6_^2−^ are anticipated. This, together with Sr/Ti = 1, is expected to provide lower supersaturation conditions compared to that formerly reported, where Sr/Ti was 12.^29,42^ Considering that the major Bi_4_Ti_3_O_12_ platelets remained undissolved, the ratio of Sr^2+^(aq) to [Ti(OH)_6_]^2−^(aq) at the beginning of the process is much higher than stoichiometric. The growth of square-like SrTiO_3_ islands on the Bi_4_Ti_3_O_12_ template platelets is evident in Fig. 4a. Compared to conditions with higher Sr/Ti ratios (Fig. 4), at Sr/Ti = 1 the SrTiO_3_ islands are larger, thicker, isolated and appear more frequently close to the edge of the basal surface plane of the Bi_4_Ti_3_O_12_ platelet (Fig. 4a and S3, ESI†). In many cases the edges of the platelets are partially or even completely rimmed by SrTiO_3_ (Fig. S3c, ESI†). The corresponding morphological development is in accordance with the atomistic observation of the Bi_4_Ti_3_O_12_ platelet's morphology, as well as with the theory of nucleation and crystal growth. As observed by STEM and SEM (Fig. 1), several steps are present at the lateral surfaces of the Bi_4_Ti_3_O_12_ platelet, while at the basal surface planes, the steps are rarely in the middle but more frequently close to the edge (Fig. 1b and c). According to classical nucleation–crystallization theory, the kink sites at the steps are the most favorable location for the incorporation and dis-incorporation of structural units.^30,43^ As a consequence the dissolution of the pseudo-perovskite [Bi_2_Ti_3_O_10_]^2−^ blocks and bismuth oxide ([Bi_2_O_2_]^2+^) layers of the Bi_4_Ti_3_O_12_ platelets proceeds faster from the lateral side and the higher population of steps with kink sites close to the edge of basal surface planes are also the most favorable sites for the beginning of SrTiO_3_ growth. Additionally, the solubility product for the SrTiO_3_ formation at these places is expected to be exceeded earlier than in the middle of the basal surface plane because the dissolution of the pseudo-perovskite [Bi_2_Ti_3_O_10_]^2−^ blocks started from the lateral side and, consequently, the areas close to the edge are at least at the beginning of the process subjected to higher [Ti(OH)_6_]^2−^(aq) concentrations. Therefore, the preferential growth of SrTiO_3_ close to the edge is also a consequence of a higher initial local supersaturation that triggers the on-set of SrTiO_3_ nucleation preferentially there. But, the SrTiO_3_ islands that are grown in lines imply the start of SrTiO_3_ growth at the kink sites in the steps, which were initially present on the basal surface plane of the Bi_4_Ti_3_O_12_ platelets (Fig. 4a). Fig. 4 clearly shows that the SrTiO_3_ coverage of the basal surface plane of Bi_4_Ti_3_O_12_ platelets increases with increments of the Sr/Ti ratio. The appearance of the SrTiO_3_ growths become more layer-by-layer-like and the individual islands are becoming less discernible at Sr/Ti = 24 (Fig. 4d and S4, ESI†). Actually, the morphological development evident from Fig. 4a–d indicates polynuclear 2D growth, which is a consequence of the high nucleation rates at sufficiently high supersaturation.^31^ Even more, the distinct increase in the number of islands with the increase of strontium (Sr/Ti ratio) is assumed to reflect the same trend in supersaturation (Fig. 4a–c). In other words, this also implies that at a defined NaOH concentration, higher initial Sr/Ti ratios could be correlated with a higher concentration of Sr^2+^ in the solution and with a higher supersaturation.

The SrTiO_3_ islands are the largest and the thickest in the case of the lowest Sr/Ti = 1, which provides the worst protection of the Bi_4_Ti_3_O_12_ basal surface plane. In addition, the system with Sr/Ti = 1 presumably in the series of Sr/Ti ratios ensures the lowest supersaturation, which is also expected to show the steepest decrease in the progress of SrTiO_3_ growth, which preferentially continues in areas already covered with SrTiO_3_. Namely, the nucleation-energy barrier (eqn (3)) is lower for the growth of SrTiO_3_ on SrTiO_3_ (no misfit, in eqn (3): *α*^3^ ∼ negligible) than for the growth of SrTiO_3_ on Bi_4_Ti_3_O_12_ (Table 2). The explanation why higher *c*_Sr^2+^_ enables better SrTiO_3_ overgrowth over the basal surface planes of the Bi_4_Ti_3_O_12_ platelet is not solely established based on the formation of the first Sr^2+^ protective layer, but a clear elucidation is also evident from eqn (3). Since it is expected that higher *c*_Sr^2+^_ ensures higher supersaturation (*S*), providing larger term (ln *S*)^2^ in the denominator of eqn (3) and consequently a lower nucleation energy barrier. As a result, an increase of the 2D nucleation rate with an increase of the Sr/Ti ratios and continued 2D polynuclear growth of SrTiO_3_ on Bi_4_Ti_3_O_12_ is evident from Fig. 4.

**Fig. 5:**
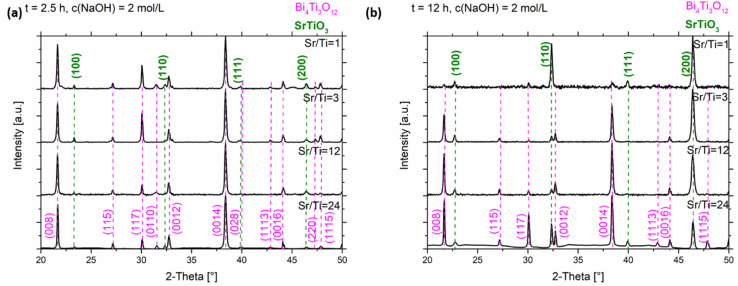
XRD patterns of the SrTiO_3_/Bi_4_Ti_3_O_12_ and SrTiO_3_ platelets (cast on single-crystalline silicon substrate) formed in 2 mol L^−1^ NaOH at 200 °C with different initial Sr/Ti ratio (a) after 2.5 hours and (b) after 12 hours.

**Fig. 6:**
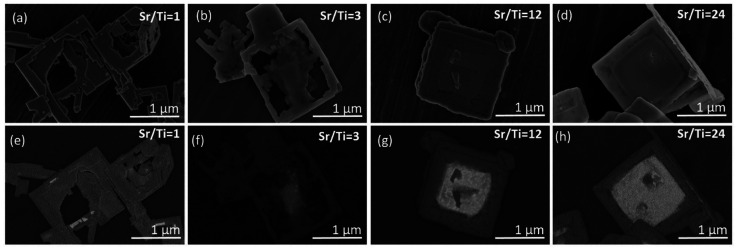
SEM micrographs of the SrTiO_3_ structures and SrTiO_3_/Bi_4_Ti_3_O_12_ platelet after 12 h reaction at 200 °C in 2 mol L^−1^ NaOH and at different Sr/Ti ratios: (a and e) Sr/Ti = 1, (b and f) Sr/Ti = 3, (c and g) Sr/Ti = 12, (d and h) Sr/Ti = 24. Secondary electron (SE) images: (a–d) and backscattered electrons (BSE) images: (e–h).

In the XRD patterns of the SrTiO_3_/Bi_4_Ti_3_O_12_ heterostructures obtained after 2.5 hours of reaction (Fig. 5a and S5a, ESI†), regardless of the initial Sr/Ti ratios, the Bi_4_Ti_3_O_12_ diffractions dominate over those of SrTiO_3_. This is according to expectations because the SrTiO_3_ layers on the Bi_4_Ti_3_O_12_ are still thin and a major part of Bi_4_Ti_3_O_12_ remained unreacted after this short time at 200 °C in 2 mol L^−1^ NaOH. A significant advance of the transformation to SrTiO_3_ was observed after a 12 hour-reaction (Fig. 5b and S5b, ESI†). Almost pure SrTiO_3_ was obtained at Sr/Ti = 1 (∼98% SrTiO_3_ and 2% Bi_4_Ti_3_O_12_ remains), whereas the amount of unreacted Bi_4_Ti_3_O_12_ increased with Sr excess (Fig. 5b). For example, the remains of Bi_4_Ti_3_O_12_ are 27% at Sr/Ti = 3 and further increases to 42% and 58% for Sr/Ti = 12 and 24, respectively. The unreacted Bi_4_Ti_3_O_12_ is clearly visible as the lighter core phase in the backscattered electron (BSE) images in Fig. 6 and S6, ESI.† These results indicate that higher Sr concentrations impede the conversion from Bi_4_Ti_3_O_12_ to SrTiO_3_. The transformation is the fastest for Sr/Ti = 1, that among the studied Sr/Ti ratios yield the lowest supersaturation, which limits the SrTiO_3_ growth predominantly in regions close to the edge (for nucleation the most favorable places), while the middle parts of the Bi_4_Ti_3_O_12_ basal surface planes remained unprotected against dissolution. Hence, the supply of [Ti(OH)_6_]^2−^(aq) is not restricted to a long diffusion path from the groove (as in the case of higher supersaturation (Fig. S1, ESI†)^29^ and the SrTiO_3_ growth continues, predominantly on the initially formed SrTiO_3_ layer around the platelet edges. The described transformation process is also supported by the formation of SrTiO_3_ frame-like platelets (Fig. 6a and S6a, ESI†). The integrity of the SrTiO_3_/Bi_4_Ti_3_O_12_ platelets after a 12-hour reaction in 2 mol L^−1^ NaOH is still impaired for Sr/Ti = 3, but much better preserved for Sr/Ti = 12 and 24 (Fig. 6 and S6, ESI†). The latter conditions enable the best preservation of morphology, but the sluggishness of the reaction significantly extends the time needed for complete conversion to SrTiO_3_ (Fig. 5b). All these observations emphasize that higher Sr concentrations (Sr/Ti = 12, 24) are needed to direct the nucleation and growth of SrTiO_3_ over the whole basal surface planes of the Bi_4_Ti_3_O_12_ platelets. When these are entirely protected by SrTiO_3_, the supply of [Ti(OH)_6_)]^2−^(aq) for further SrTiO_3_ growth is limited by the dissolution of pseudo-perovskite blocks from the groove, which deepens with the progress of the reaction and consequently the reaction is slowed down.

The above-described growths of (100) SrTiO_3_ on the (001) Bi_4_Ti_3_O_12_ platelets in some respects resemble the epitaxial growth of thin films on chemically different, dissimilar crystalline substrates, where the structural matching and supersaturation play a key role and determine the type of growth mode. The latter, depending on the lattice mismatch, varies from layer-by-layer growth for a negligible misfit (Frank–van der Merwe) to layer-plus-island growth (Stranski–Krastanov) or island growth (Volmer–Weber) for a perceptible misfit.^30^ Our results clearly demonstrate that conditions with Sr/Ti ≥ 12 are capable of balancing the 1.4% –2.1% misfit (Table 2) and direct the SrTiO_3_ growth over the whole basal surface planes of Bi_4_Ti_3_O_12_ and consequently ensure the preservation of the platelet morphology through the process.

#### 3.3.2. Transformation control at a higher base concentration (6 mol L^−1^ NaOH)

**Fig. 7:**
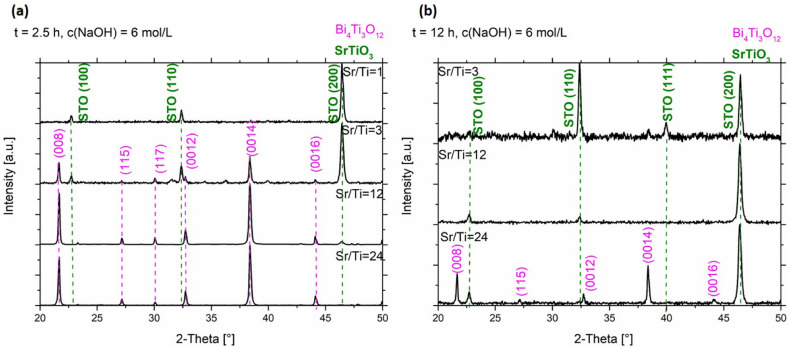
XRD patterns of the SrTiO_3_/Bi_4_Ti_3_O_12_ and SrTiO_3_ platelets (cast on single-crystalline silicon substrate) formed in 6 mol L^−1^ NaOH at 200 °C with different initial Sr/Ti ratio (a) after 2.5 hours and (b) after 12 hours.

To achieve a complete transformation in a reasonable time, the dissolution of pseudo-perovskite blocks has to be accelerated. In view of reaction 1, this can be realized by a higher concentration of base (NaOH). Bi_4_Ti_3_O_12_ is therefore expected to dissolve more and faster in 6 mol L^−1^ NaOH, providing higher concentrations of [Ti(OH)_6_]^2−^(aq) compared to that in 2 mol L^−1^ NaOH. Nevertheless, the Bi_4_Ti_3_O_12_ dissolution should still be slow enough that the platelets can still serve as the substrate for the epitaxial growth of SrTiO_3_. A qualitative evaluation of the stability of the Bi_4_Ti_3_O_12_ platelets in 6 mol L^−1^ NaOH at 200 °C revealed no drastic change in the general dimensions of the platelets, even after a 15 hour treatment under such conditions.^29^ Nevertheless, according to the STEM observation hot NaOH causes a relatively rapid removal of the [Bi_2_O_2_]^2+^ termination layer (Fig. S2, ESI†). Similar to 2 mol L^−1^ NaOH, the transformation rate from Bi_4_Ti_3_O_12_ to SrTiO_3_ and the accompanying morphological development were also systematically examined in 6 mol L^−1^ NaOH and at various initial Sr/Ti ratios (*i.e.*, Sr/Ti = 1, 3, 12, 24). A comparison of the XRD patterns, shown in Fig. 5 and 7 (Fig. S5 and S7, ESI†), revealed that for a particular Sr/Ti ratio the transformation to SrTiO_3_ is much faster for reactions performed in more concentrated NaOH solutions. For example, in 6 mol L^−1^ NaOH (200 °C) for the system with initial Sr/Ti = 1, the complete transformation to SrTiO_3_ occurs in less than 2.5 hours (Fig. 7a and S7a, ESI†), while approximately 12 hours is needed for the initial Sr/Ti = 12 (Fig. 7b and S7b, ESI†). However, for initial Sr/Ti = 24, a considerable amount (20%) of Bi_4_Ti_3_O_12_ remained untransformed (SrTiO_3_/Bi_4_Ti_3_O_12_) after a 12-hour reaction under the same conditions (6 mol L^−1^ NaOH, 200 °C) (Fig. 7b, S7b and S8, ESI†).

**Fig. 8:**
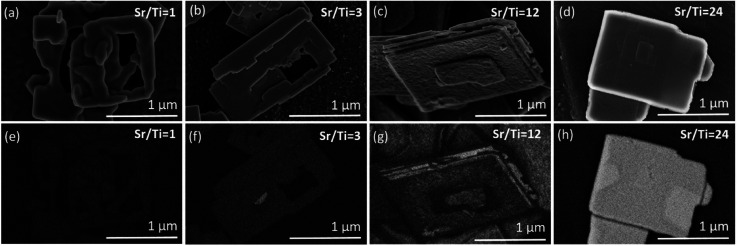
SEM micrographs of SrTiO_3_ and SrTiO_3_/Bi_4_Ti_3_O_12_ platelets after 2.5 hours at 200 °C in 6 mol L^−1^ NaOH at different initial Sr/Ti ratios: (a and e) Sr/Ti = 1, (b and f) Sr/Ti = 3, (c and g) Sr/Ti = 12, (d and h) Sr/Ti = 24. (SE images: (a–d) and BSE images: (e–h)).

**Fig. 9:**
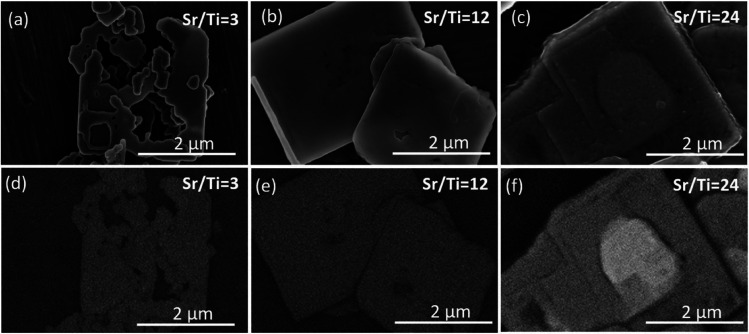
SEM micrographs of SrTiO_3_ and SrTiO_3_/Bi_4_Ti_3_O_12_ platelets after 12 hours at 200 °C in 6 mol L^−1^ NaOH at different initial Sr/Ti ratios: (a and d) Sr/Ti = 3, (b and e) Sr/Ti = 12, (c and f) Sr/Ti = 24. (SE images: (a–c) and BSE images: (d–f)).

In the series of Sr/Ti ratios, the experimental conditions with Sr/Ti = 1 also presumably provide in 6 mol L^−1^ NaOH the lowest supersaturation, a decrease of which with the progress of SrTiO_3_ formation is more notable than for those with higher Sr/Ti ratios. This reflects in the morphology of the formed SrTiO_3_ particles (nanostructures), which at Sr/Ti = 1 in some cases considerably deviate from the initial template (Fig. 8a). Nanocubes, nanoblocks and various plate-like irregular morphologies were observed. It must be mentioned that in none of these SrTiO_3_ nanostructures was the characteristic groove observed, indicating that the growth of SrTiO_3_ does not occur simultaneously on both basal surface planes of the Bi_4_Ti_3_O_12_, and the dissolution is also not restricted exclusively from the lateral side. This means that SrTiO_3_ continues to grow on initially nucleated SrTiO_3_ regions on the Bi_4_Ti_3_O_12_ platelet, while unprotected areas of the template dissolve. Similar to the 2 mol L^−1^-NaOH-experiments, also under higher base concentrations, better SrTiO_3_ coverage and protection of the basal surface planes (Fig. 8 and 9) were observed at conditions with higher Sr/Ti ratios.

### 3.4. Formulation of the growth mechanism

The above results, presented in Fig. 4, 6, 8 and 9, demonstrate that when the transformation from Bi_4_Ti_3_O_12_ to SrTiO_3_ is performed in alkaline (2 mol L^−1^ or 6 mol L^−1^ NaOH) aqueous solutions at 200 °C with Sr/Ti ≥ 12, the intermediate SrTiO_3_/Bi_4_Ti_3_O_12_ and final SrTiO_3_ structures could well maintain the platelet morphology of the initial Bi_4_Ti_3_O_12_ template. The conditions with higher Sr/Ti ratios enable better protection of the basal surface planes against degradation from the top and the SrTiO_3_ nucleation and growth occur over the entire basal surface planes, while the Bi_4_Ti_3_O_12_ dissolution proceeds from the lateral side. The process of Bi_4_Ti_3_O_12_ dissolution and SrTiO_3_ epitaxial growth continues until the complete transformation and formation of SrTiO_3_ nanoplatelets, that consist of two parallel, partially intergrown platelets. The groove that extends in the middle and parallel of the platelet's basal surface plane is the outcome of the Bi_4_Ti_3_O_12_ dissolution from the lateral side (Fig. S1, ESI†). The combination of high-quality template nanoplatelets, Sr/Ti = 12 and 6 mol L^−1^ NaOH, provides the conditions for the complete transformation of Bi_4_Ti_3_O_12_ into SrTiO_3_ nanoplatelets in a reasonably short synthesis time of less than 12 hours (Fig. 9b and S8, ESI†). The above results shed light on the interplay of interfacial free energy and supersaturation in the topotactic epitaxy of SrTiO_3_ on Bi_4_Ti_3_O_12_. In addition to a theoretical understanding of the mechanism, the study also provides empirical guidelines to ensure that intermediate SrTiO_3_/Bi_4_Ti_3_O_12_ and final SrTiO_3_ retain the platelet morphology of the template. The presented procedure can also serve a general strategy for controlling hydrothermal transformations that proceed through dissolution and epitaxial growth processes.

## 4. Conclusions

Engineering of particulate 2D epitaxial heterostructures and their formation mechanisms are not well addressed in the literature, despite the expected outstanding functional properties of these materials. In the present study, the exploitation of the hydrothermal topotactic epitaxy approach to the formation of 2D epitaxial heterostructures and anisotropic 2D structures is exemplified by the detailed mechanistic study of the hydrothermal topochemical transformation of Bi_4_Ti_3_O_12_ platelets to intermediate SrTiO_3_/Bi_4_Ti_3_O_12_ and final SrTiO_3_ platelets. The atomic scale insight into the microstructures of initial Bi_4_Ti_3_O_12_ and the reaction products at different transformation stages, under different experimental conditions enabled us to completely understand the transformation process in terms of nucleation–crystallization theory. The SrTiO_3_ grows epitaxially in the (100) orientation over the (001)-oriented Bi_4_Ti_3_O_12_ platelets, whereby the misfit between the relevant pairs of lattice planes is manifested by the formation of linear dislocations at the interface. The key experimental parameters of the transformation process at 200 °C, which determine the kinetics and the path of the reaction as well as the morphology of the intermediate and final products are Sr content (Sr/Ti ratio) and base (NaOH) concentration. A large excess of Sr (Sr/Ti ≥ 12) leads to a high nucleation rate that is continued by 2D polynuclear SrTiO_3_ growth. As a consequence, this initially rapid SrTiO_3_ overgrowth of the Bi_4_Ti_3_O_12_ basal surface planes protects the platelets' dissolution from the top and thus ensures that the intermediate SrTiO_3_/Bi_4_Ti_3_O_12_ and final SrTiO_3_ platelets retain the morphology of the initial template. At a certain Sr/Ti initial molar ratio, the rate of SrTiO_3_ formation is governed by the base concentration. Higher base concentrations accelerate the dissolution of Bi_4_Ti_3_O_12_ and consequently the SrTiO_3_ formation.

A thorough understanding of this transformation process establishes the guidelines for the engineering of other epitaxial heterostructures or defined (anisotropic) nanostructures *via* hydrothermal topotactic epitaxy. In the engineering of this kind of reaction many parameters must be matched, such as a sufficient solubility/stability of phases in hydrothermal media, phases in crystallographic orientations with low lattice mismatch and appropriately defined morphology. Nevertheless, this approach provides ideas for designing several new particle heterostructures with ordered epitaxial interfaces or the preparation of anisotropic 1D or 2D nanostructures or other particle architectures at low synthesis temperatures.

## Conflicts of interest

There are no known conflicts of interest to declare.

## Supplementary Material

NA-005-D2NA00741J-s001
