# Supplementary material for: Hydrothermal topotactic epitaxy of SrTiO3 on Bi4Ti3O12 nanoplatelets: understanding the interplay of lattice mismatch and supersaturation
Source: Nanoscale Adv. 2023 May 8;5(11):3005–17. doi: 10.1039/d2na00741j (PMC10228367; doi:10.1039/d2na00741j)
Supplement: NA-005-D2NA00741J-s001 [file NA-005-D2NA00741J-s001.pdf]

Electronic Supplementary Material (ESI) for Nanoscale Advances.
This journal is © The Royal Society of Chemistry 2023

# Electronic Supplementary Information

## Hydrothermal topotactic epitaxy of $SrTiO_3$ on $Bi_4Ti_3O_{12}$ nanoplatelets: understanding the interplay of lattice mismatch and supersaturation

Alja Čontala, Nina Daneu, Suraj Gupta, Matjaž Spreitzer, Anton Meden and Marjeta Maček Kržmanc*

**Table S1:** Final concentrations (c) of reagents before hydrothermal reaction at 200 °C

| $c_{Bi4Ti3O12}$ (mol/l) | Initial Sr/Ti ratio | $c_{SrCl2x6H2O}$ (mol/L) | $c_{NaOH}$ (mol/L) |
|---|---|---|---|
| 0.00102 | 1:1 | 0.00306 | 2 and 6 |
| | 3:1 | 0.00918 | |
| | 12:1 | 0.03672 | |
| | 24:1 | 0.07344 | |

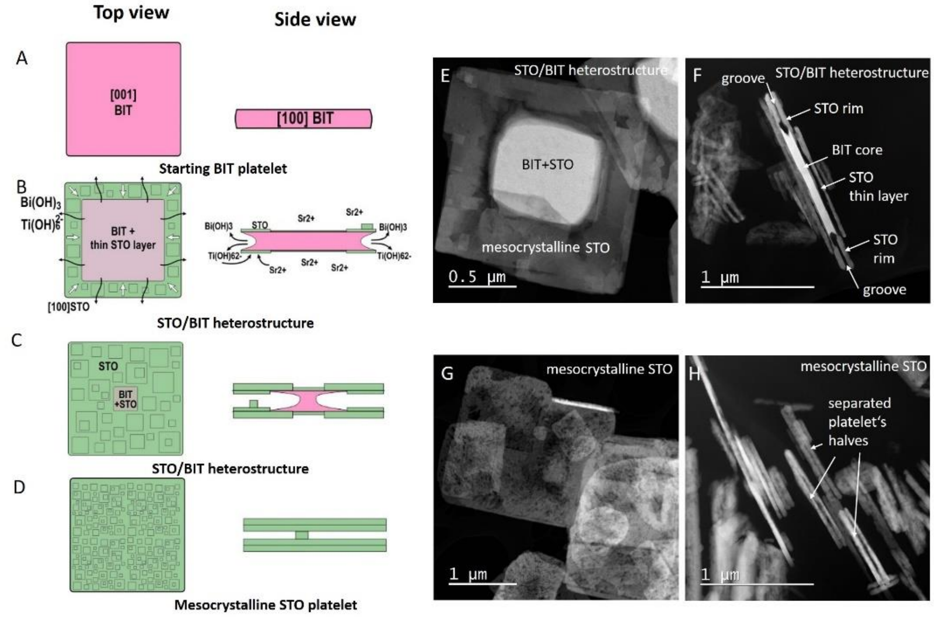


**Figure S1:** (A-D) schematic presentation of the $Bi_4Ti_3O_{12}$-to-$SrTiO_3$ transformation from the top and side view: (A) ⇒initial $Bi_4Ti_3O_{12}$ (BIT) platelets, (B, C)⇒$SrTiO_3/Bi_4Ti_3O_{12}$ (STO/BIT) heterostructural platelets at different stages of the transformation, (D) $SrTiO_3$ (STO) platelets. (E-H) STEM micrographs of the (E, F) heterostructural $SrTiO_3/Bi_4Ti_3O_{12}$ (STO/BIT) platelets and (G,H) $SrTiO_3$ platelets from the top (E, G) and side view (F,H). The STEM micrographs, shown in E and F, and those presented in G and H, correspond to the to the transformation schematically presented at B and D, respectively.[1]

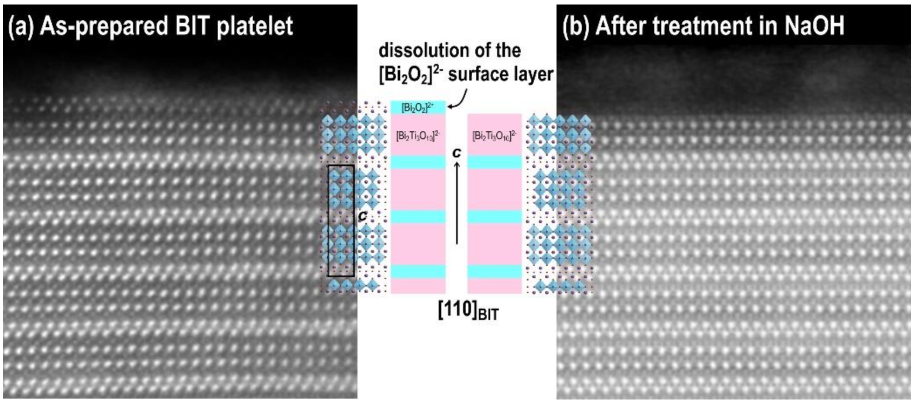


**Figure S2**: Cross-sectional HAADF-HR-STEM images of the $Bi_4Ti_3O_{12}$ (BIT) platelet in the [110] orientation taken near the surface of the platelets with overlaid structural models: (a) As-prepared $Bi_4Ti_3O_{12}$ (BIT) platelets, terminated by bismuth oxide $[Bi_2O_2]^{2-}$ layer (the black rectangular denotes the unit cell of $Bi_4Ti_3O_{12}$) and (b) $Bi_4Ti_3O_{12}$ (BIT) platelets after exposure to hot NaOH (6 mol/L NaOH, holding time 1 hour at 200 °C), terminated by pseudoperovskite ($[Bi_2Ti_3O_{10}]^{2-}$) blocks.

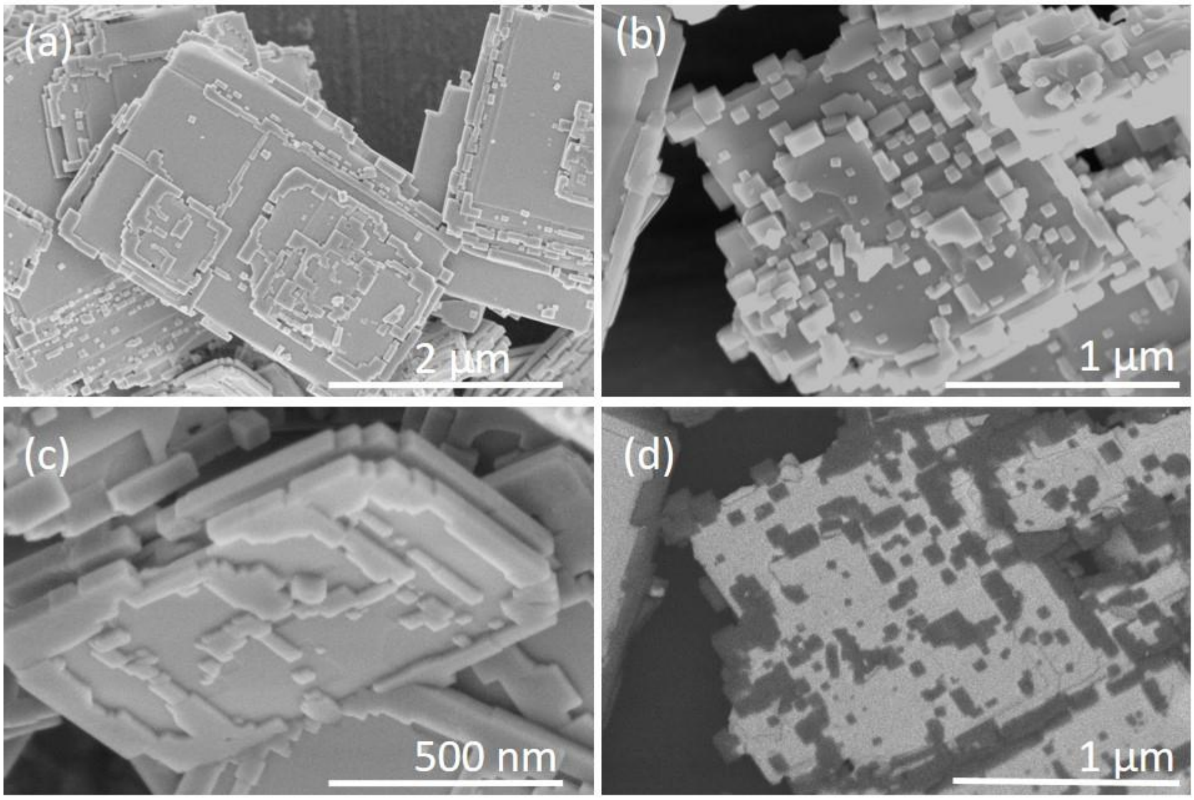


**Figure S3**: SEM micrographs (**a-c** secondary electron (SE) images) of the $SrTiO_3/Bi_4Ti_3O_{12}$ platelets after 2.5 h at 200 °C at Sr/Ti=1 in 2 mol/l NaOH. Image (**d**) is backscattered (BSE) image of the image shown in (**b**).

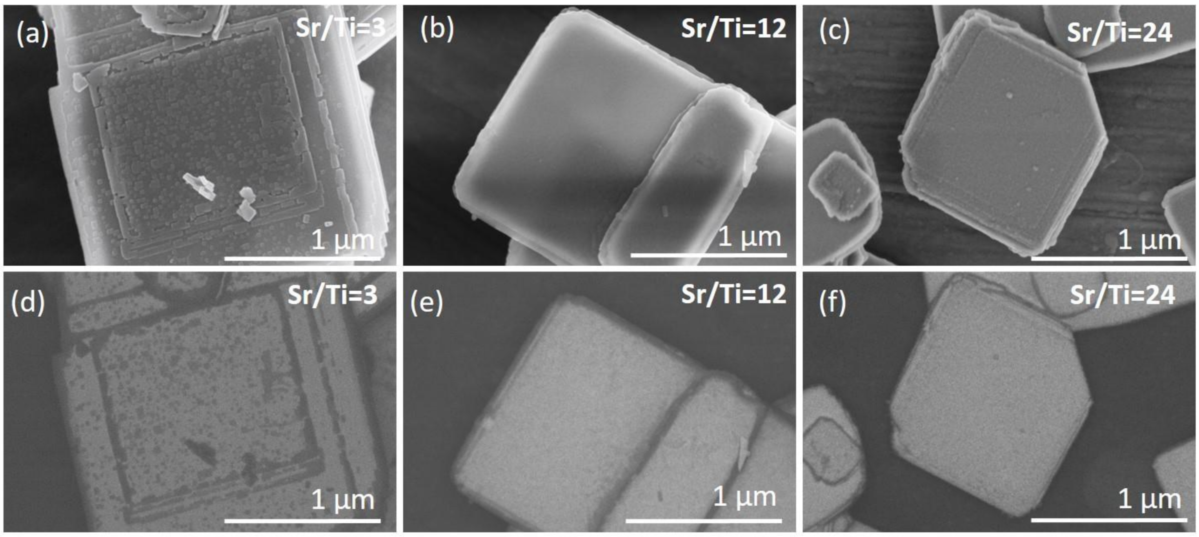


**Figure S4**: SEM micrographs (SE images: **a,b,c** and BSE images: **d,e,f**) of the $SrTiO_3/Bi_4Ti_3O_{12}$ platelets after 2.5 h at 200 °C in 2 mol/l NaOH at various Sr/Ti ratios: (**a, d**) Sr/Ti=3, (**b, e**) Sr/Ti=12, (**c, f**) Sr/Ti=24.

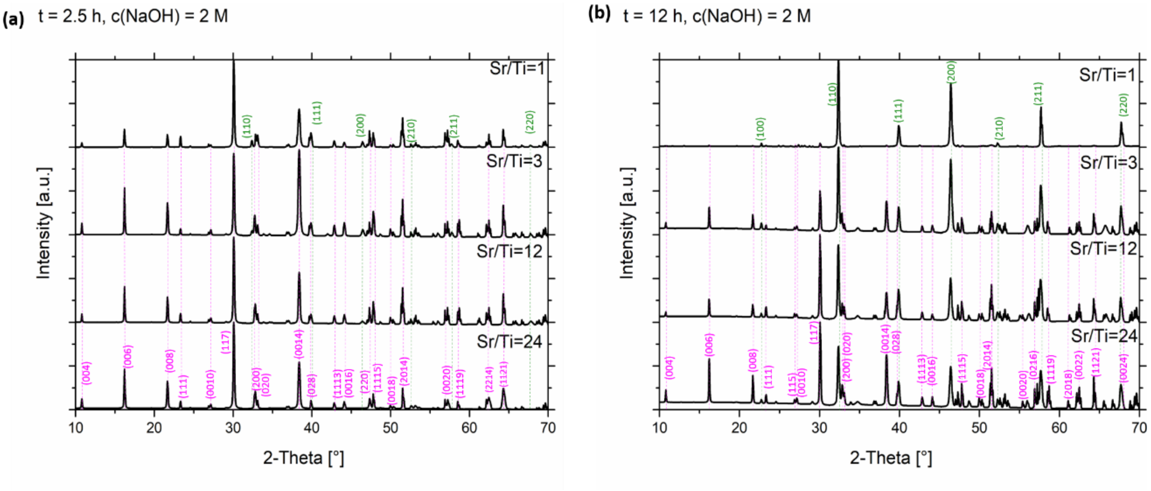


**Figure S5**: Powder XRD patterns ($2\theta$: 10 °-70 °) of $SrTiO_3/Bi_4Ti_3O_{12}$ and $SrTiO_3$ reaction product after 2.5 h and 1 2h at 200 °C in 2 mol/L NaOH at different Sr/Ti ratios. The XRD patterns were indexed with JCPDS reference cards 01-074-1296 and 01-072-1019 for $SrTiO_3$ (STO) and $Bi_4Ti_3O_{12}$ (BIT), respectively.

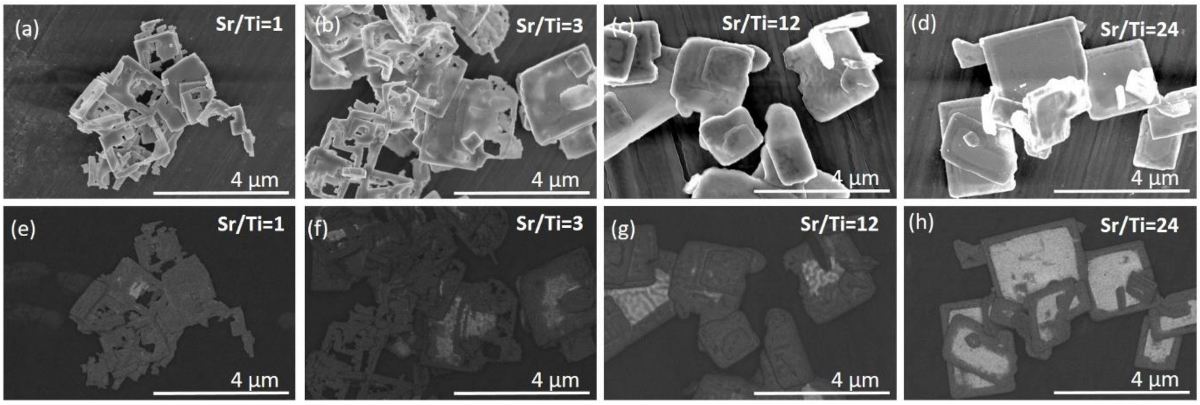


**Figure S6:** SEM micrographs of the $SrTiO_3$ structures and $SrTiO_3/Bi_4Ti_3O_{12}$ platelet after 12 h reaction at 200 °C in 2 mol/L NaOH and at different Sr/Ti ratios (**a-d**: SE images, **e-h:** corresponding BSE images).

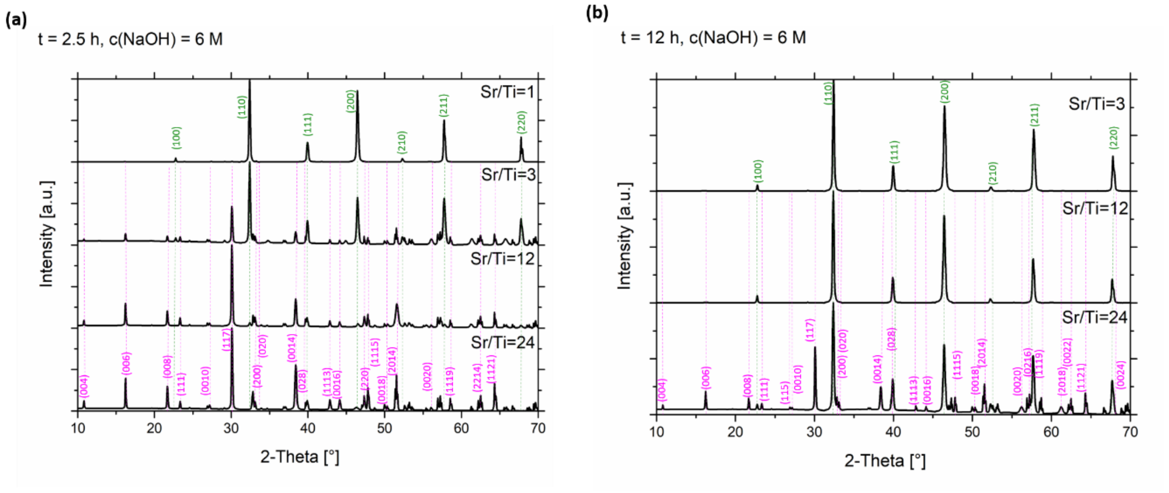


**Figure S7**: Powder XRD patterns (2θ: 10 °-70 °) of $SrTiO_3/Bi_4Ti_3O_{12}$ and $SrTiO_3$ reaction product after 2.5 h and 12 h at 200 °C in 6 mol/L NaOH at different Sr/Ti ratios. The XRD patterns were indexed with JCPDS reference cards 01-074-1296 and 01-072-1019 for $SrTiO_3$ (STO) and $Bi_4Ti_3O_{12}$ (BIT), respectively.

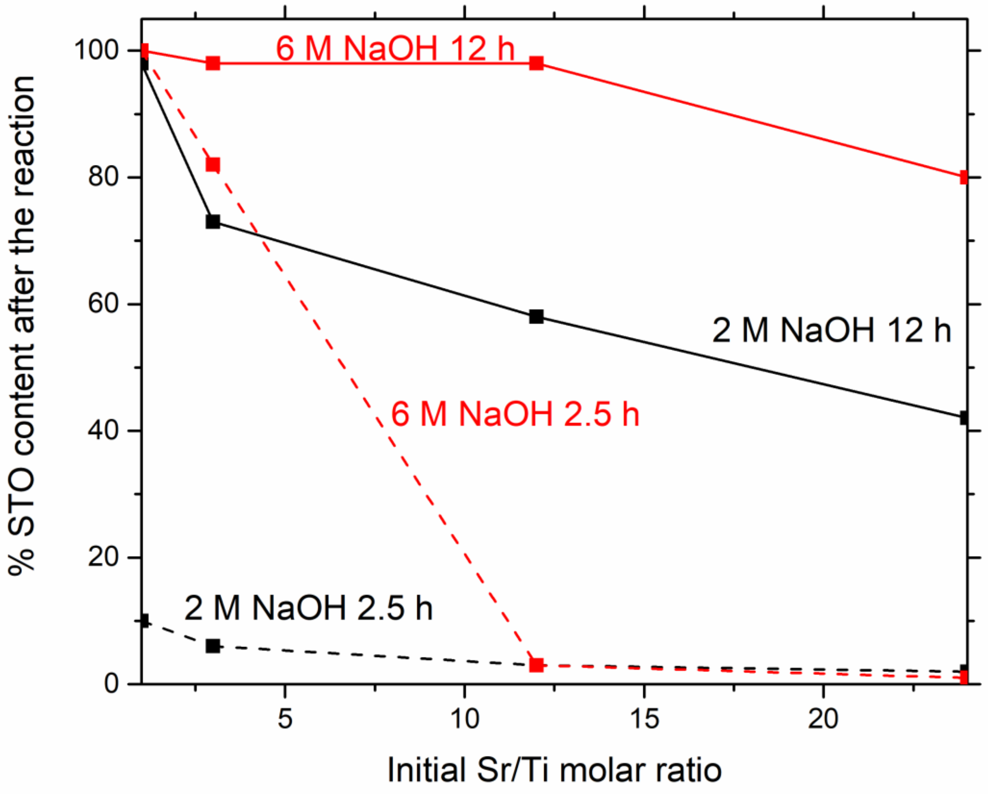


**Figure S8:** Graphical presentation of the $SrTiO_3$ (STO) content in percentage after the reaction. The lines are added for visualisation; however, the reactions were performed only for Sr/Ti=1, 3, 12 and 24 molar ratios (the points denoted with squares). The black lines are for the reactions in 2 mol/L NaOH and the red lines are for the reactions in 6 mol/L NaOH. In both cases, dashed lines are for the reactions after 2.5 h and solid for the reactions after 12 h. All reactions were performed at 200 °C.

## References


(1) Maček Kržmanc, M.; Daneu, N.; Čontala, A.; Santra, S.; Kamal, K. M.; Likozar, B.; Spreitzer, M. $SrTiO_3/Bi_4Ti_3O_{12}$Nanoheterostructural Platelets Synthesized by Topotactic Epitaxy as Effective Noble-Metal-Free Photocatalysts for PH-Neutral Hydrogen Evolution. *ACS Appl. Mater. Interfaces* **2021**, *13* (1), 370–381. https://doi.org/10.1021/acsami.0c16253.
